# Long noncoding RNAs in *Brassica rapa* L. following vernalization

**DOI:** 10.1038/s41598-019-45650-w

**Published:** 2019-06-26

**Authors:** Daniel J. Shea, Namiko Nishida, Satoko Takada, Etsuko Itabashi, Satoshi Takahashi, Ayasha Akter, Naomi Miyaji, Kenji Osabe, Hasan Mehraj, Motoki Shimizu, Motoaki Seki, Tomohiro Kakizaki, Keiichi Okazaki, Elizabeth S. Dennis, Ryo Fujimoto

**Affiliations:** 1 Graduate School of Science and Technology, Niigata University, Ikarashi-ninocho, Niigata, 950-2181 Japan; 2 Graduate School of Agricultural Science, Kobe University, Rokkodai, Nada-ku, Kobe 657-8501 Japan; 3 Institute of Vegetable and Floriculture Science, NARO, Kusawa, Ano, Tsu, Mie 514-2392 Japan; 4 RIKEN Center for Sustainable Resource Science, Yokohama, Kanagawa 230-0045 Japan; 5 Plant Epigenetics Unit, Okinawa Institute of Science and Technology Graduate University, Onna-son, Okinawa 904-0495 Japan; 6 Iwate Biotechnology Research Center, Narita, Kitakami, Iwate, 024-0003 Japan; 7 Core Research for Evolutional Science and Technology, Japan Science and Technology, Kawaguchi Saitama, 332-0012 Japan; 8 RIKEN Cluster for Pioneering Research, 2-1 Hirosawa, Wako, Saitama 351-0198 Japan; 9 CSIRO Agriculture and Food, Canberra, ACT 2601 Australia; 10 University of Technology, Sydney, PO Box 123, Broadway, NSW 2007 Australia

**Keywords:** Gene expression, Plant molecular biology

## Abstract

*Brassica rapa* L. is an important agricultural crop that requires a period of prolonged cold for flowering. This process is known as vernalization. Studies have shown that long noncoding RNAs (lncRNAs) play important roles in abiotic stress responses and several cold-responsive noncoding RNAs have been suggested to be involved in vernalization. We examined the transcriptome of the Chinese cabbage inbred line (*B. rapa* L. var. *pekinensis*) RJKB-T24, and identified 1,444 long intergenic noncoding RNAs (lincRNAs), 551 natural antisense transcripts (NATs), and 93 intronic noncoding RNAs (incRNAs); 549 of the 2,088 lncRNAs significantly altered their expression in response to four weeks of cold treatment. Most differentially expressed lncRNAs did not lead to a change of expression levels in mRNAs covering or near lncRNAs, suggesting that the transcriptional responses to four weeks of cold treatment in lncRNA and mRNA are independent. However, some differentially expressed mRNAs had NATs with expression altered in the same direction. These genes were categorized as having an abiotic stress response, suggesting that the paired-expression may play a role in the transcriptional response to vernalization or cold treatment. We also identified short-term cold treatment induced NATs in *BrFLC* and *BrMAF* genes, which are involved in vernalization. The lncRNAs we identified differed from those reported in *Arabidopsis thaliana*, suggesting the role of lncRNAs in vernalization differ between these two species.

## Introduction

Long noncoding RNAs (lncRNAs) are longer than 200 nucleotides (nt) in contrast to small RNAs (18 to 30 nt in length) such as microRNAs (miRNAs) or small interfering RNAs (siRNAs). LncRNAs are classified into three major groups, long intergenic noncoding RNAs (lincRNAs), intronic noncoding RNAs (incRNAs) derived from introns, and natural antisense transcripts (NATs) transcribed from the complementary DNA strand of their associated genes^1,2^. RNA-sequencing (RNA-seq) enables the identification of novel transcripts including lncRNAs at the whole genome level in many plant species^1,2^. Generally, lncRNAs show low expression levels and have low sequence conservation even between closely related species^1,2^. Therefore, while many lncRNAs have been identified, few have had their biological function clarified^1,2^. LncRNAs have been shown to play important roles in vernalization^3–5^, photoperiod-sensitive male sterility^6^, red-light-mediated seedling photomorphogenesis^7^, seed dormancy^8^, and the transcriptional regulation of plant innate immunity^9^.

In *Arabidopsis thaliana*, the *FLOWERING LOCUS C* (*FLC*) gene encodes a MADS box transcription factor, and is a key gene in the vernalization process^10,11^. *FLC* is expressed before prolonged cold exposure and acts as a repressor of flowering^10,11^. During prolonged cold exposure, trimethylation of lysine 27 on histone H3 (H3K27me3) accumulates at the *FLC* locus maintaining the *FLC* gene in the repressed state^10–13^. This accumulation of H3K27me3 is mediated by the PHD-PRC2 (Plant homeodimain-Polycomb Repressive Complex 2) complex^10–13^. Three cold-responsive noncoding RNAs (COOLAIR^3^, COLDAIR^4^, and COLDWRAP^5^) derived from different regions within the *FLC* locus, have been suggested to be involved in *FLC* silencing or in maintenance of the *FLC* repressed state^10,14^. The FLC protein belongs to a MADS-box protein family, which contains five other members, MADS AFFECTING FLOWERING 1–5 (MAF1-MAF5). *MAF1*-*MAF4* have been reported to be repressed following vernalization, and this repression is associated with an increase of H3K27me3 at the *MAF1*-*MAF4* loci^15^.

*B. rapa* comprises commercially important vegetable crops consumed worldwide, including leafy vegetables such as Chinese cabbage (var. *pekinensis*), pak choi (var. *chinensis*), and komatsuna (var. *perviridis*), and root vegetables including turnip (var. *rapa*)^16^. For leafy vegetable cultivars, bolting before plants reach the harvesting stage markedly impairs their product value, thus a high bolting resistance is an important trait^11,17^. Flowering (bolting) is controlled by internal (endogenous cues) and external (environmental stimuli) factors such as day length (photoperiodism) and temperature^17^. *B. rapa* is a facultative long-day (LD) plant; LD photoperiod conditions accelerate its flowering and requires prolonged cold exposure (vernalization) for flowering^17^.

In *B. rapa*, there are four *FLC* paralogs, and three of these *FLC* paralogs function as floral repressors^11,17^. The expression of all *FLC* paralogs is down-regulated by prolonged cold treatment, and H3K27me3 accumulates at each *FLC* locus in response to prolonged cold^18^. How noncoding RNAs may be involved in this process is currently unknown, although cold induced NATs have been detected at the *FLC2* locus of *B. rapa*^19^. We characterized lncRNAs at the whole genome level in *B. rapa* and compared the expression levels of the lncRNAs before and after four weeks of cold treatment. We identified short-term cold treatment induced NATs at the *BrFLC2* and *BrMAF* loci. We provide evidence of altered transcriptional patterns of lncRNA in response to four weeks of cold treatment in *B. rapa* and discuss the possible biological function of this altered expression, including whether lncRNA contributes to *FLC* suppression by prolonged cold treatment.

## Methods

### Plant materials and growth conditions

Chinese cabbage inbred line (*B. rapa* var. *pekinensis*), RJKB-T24, was used^20^. Seeds were surface sterilized and grown on agar solidified Murashige and Skoog (MS) plates with 1% (w/v) sucrose under long day (LD) condition (16 h light/8 h dark) at 21 °C. First and second leaves were harvested from 14-day-old non-vernalized plants. For vernalizing cold treatments, 14-day seedlings were treated for two, four, or six weeks at 4 °C under LD condition or six weeks of cold treatment followed by seven days under normal growth condition. After these treatments, first and second leaves were harvested from vernalized plants.

### RNA-sequencing (RNA-seq)

Total RNA from the first and second leaves, with and without cold treatments, were isolated by SV Total RNA Isolation System (Promega Co., WI, USA). We prepared two sequence libraries for RNA-seq with two replicates; (1) NV, non-vernalized samples of first and second leaves from RJKB-T24 (read length; 100 bp, paired-end sequencing (PE)); (2) V, four weeks vernalized samples of first and second leaves from RJKB-T24 (read length; 100 bp, PE). For making libraries for RNA-seq, mRNAs and noncoding RNAs were fragmented into short fragments (about 200~500nt), then the first-strand cDNA was synthesized by random hexamer-primer using the fragments as templates, and dTTP is substituted by dUTP during the synthesis of the second strand. Short fragments were purified and resolved with EB buffer for end reparation and single nucleotide A (adenine) addition. After that, the short fragments were connected with adapters, then the second strand was degraded using UNG (Uracil-N-Glycosylase). After agarose gel electrophoresis, suitable fragments were selected for PCR amplification as templates. During the QC steps, Agilent 2100 Bioanaylzer and ABI StepOnePlus Real-Time PCR System were used in the quantification and qualification of the sample libraries. The libraries were then sequenced using an Illumina HiSeqTM 2000 (100 bp, PE). Two samples of non-vernalized (NV-1 and -2) and vernalized (V-1 and -2) were used for RNA-seq as replicates and there was high correlation between replicates (Supplementary Fig. 1). 89.5 M, 63.9 M, 77.1 M, and 63.3 M clean reads were obtained from NV-1, NV-2, V-1, and V-2, respectively (Supplementary Table 1). More than 44% of reads were mapped to the reference genome (Supplementary Table 1). The data have been deposited with links to SRA accession number SRP156464 in the NCBI SRA database (https://www.ncbi.nlm.nih.gov/sra/).

### Steps of the identification of lncRNAs

**Figure 1:**
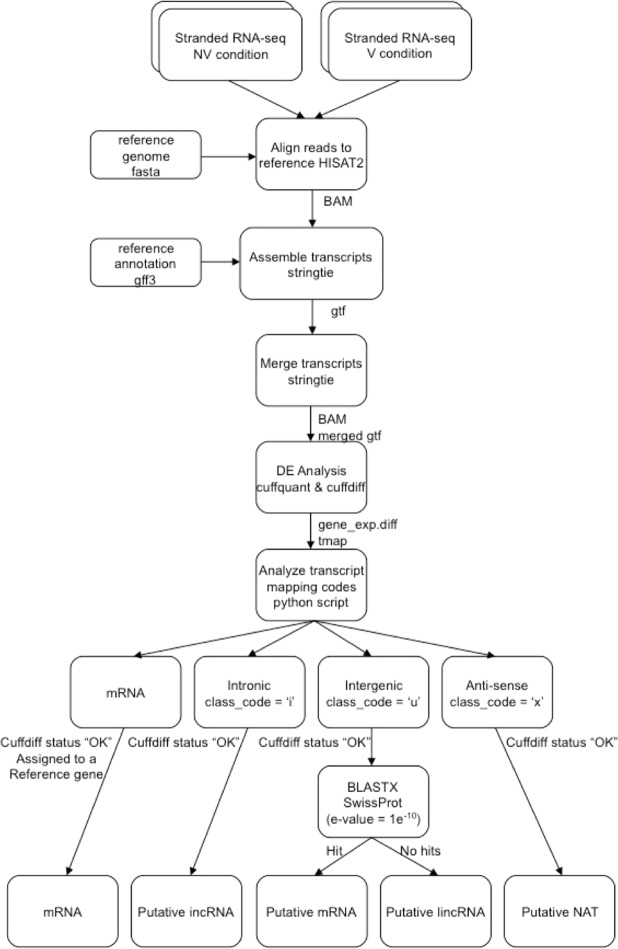
Schematic representation of the classification strategy used in this study to identify lncRNAs from RNA-seq data.

The schematic view of the steps used in the identification of lncRNAs, and the classification of the types of lncRNAs is shown in Fig. 1. To identify lncRNA transcripts, the mapping class codes, obtained from the stringtie assembly, for each transcript were examined using a custom python script (available at http://www.github.com/danshea/lncRNA). Transcripts were classified in the following manner, transcripts that matched the reference annotation and had a differential expression analysis status of “OK”, as determined by cuffdiff, were classified as mRNAs. Transcripts with a mapping class code of “x” and a differential expression analysis status of “OK” were classified as putative NATs. Transcripts with a mapping class code of “i” having a differential expression analysis status of “OK” were classified as putative incRNAs. For transcripts with a mapping class code of “u” and a differential expression analysis status of “OK”, the transcripts were further filtered by BLASTX analysis against Swissprot (e-value = 1e-10). Those transcripts matching a Swissprot entry were classified as putative unannotated genes. Transcripts that did not match any genes in the Swissprot database were then classified as putative lincRNAs.

In total, 2,052 transcripts mapped to intergenic regions of the genome and were in sufficient quantities to perform differential expression analysis by cuffquant and cuffdiff analysis. Analysis of these transcripts against the Swissprot database using BLASTX revealed that 1,444 transcripts had no hits, and 608 transcripts had hits (e-value = 1e-10). These transcripts were then assigned as lincRNAs and putative mRNAs, respectively. For NATs, 551 transcripts were mapped to the anti-sense strand of a known gene locus and were in sufficient quantities. 93 transcripts mapped to intronic regions of the genome and were in sufficient quantities, assigned as incRNAs.

### Detection of differentially expressed genes (DEGs) and lncRNAs

HISAT2^21^ was used to align RNA-seq reads against the *B. rapa* reference genome downloaded from the Brassica database (http://brassicadb.org/brad/datasets/pub/Genomes/Brassica_rapa/V1.0/V1.5/) with the following options -rna-strandness RF,--dta,--no-mixed,--no-discordant. The resultant BAM file was then sorted using samtools^22^ and the transcript assembly was performed using stringtie^23^. Gene and lncRNA expression levels were scored by fragments per kilo-base per million (FPKM) using cuffquant, and cuffdiff was used for the identification of differentially expressed genes (DEGs) and lncRNAs with and without four weeks of cold treatment^24^.

Analysis for enrichment of gene functional ontology terms was completed using the gene ontology (GO) tool, agriGO^25^ following the methods described by Shimizu *et al*. (2014)^26^. Statistical tests for enrichment of functional terms used the hypergeometric test and false discovery rate (FDR) correction for multiple testing to a level of 5% FDR.

### Gene expression analysis

To analyze gene or lncRNA expression, cDNA was synthesized from 500 ng total RNA using PrimeScript RT reagent Kit (Takara Bio., Shiga JAPAN). Prior to quantitative real-time RT-PCR (qPCR), the specificity of the primer set for each gene or lncRNA was first tested by electrophoresis of PCR amplified products using QuickTaq®HS DyeMix (TOYOBO Co., Ltd., Osaka JAPAN) on 2.0% agarose gel in which single products were observed. The absence of genomic DNA contamination was confirmed by PCR of a no-RT control. The PCR conditions were 94 °C for 2 min followed by 35 cycles of 94 °C for 30 s, 55 °C for 30 s, and 68 °C for 30 s.

qPCR was performed using a LightCycler 96 (Roche Molecular Systems, Inc., CA, USA). cDNA was amplified using FastStart Essential DNA Green Master (Roche). PCR conditions were 95 °C for 10 min followed by 40 cycles of 95 °C for 10 s, 60 °C for 10 s, and 72 °C for 10 s, and Melting program (65 °C to 97 °C at 0.1 °C/s). After amplification cycles, each reaction was subjected to melt temperature analysis to confirm single amplified products. The relative expression level of each gene or lncRNA relative to *ACTIN* (*Bractin*)^27^ was automatically calculated using automatic CQ calling according to the manufacturer’s instructions (Roche).

The expression levels of eleven genes (five up-regulated and six down-regulated genes in V) were verified by qPCR. A high correlation (*r* = 0.99) between ratio of expression levels between NV and V in these eleven genes was observed between qPCR analysis and RNA-seq data (Supplementary Fig. 2).

Data presented are the average and standard error of three biological and experimental replicates and statistically analyzed using the Student’s *t*-test, *p* < 0.05. The primers used in this study are listed in Supplementary Table 2.

## Results

### Identification of differentially expressed lncRNAs after vernalization

**Figure 2:**
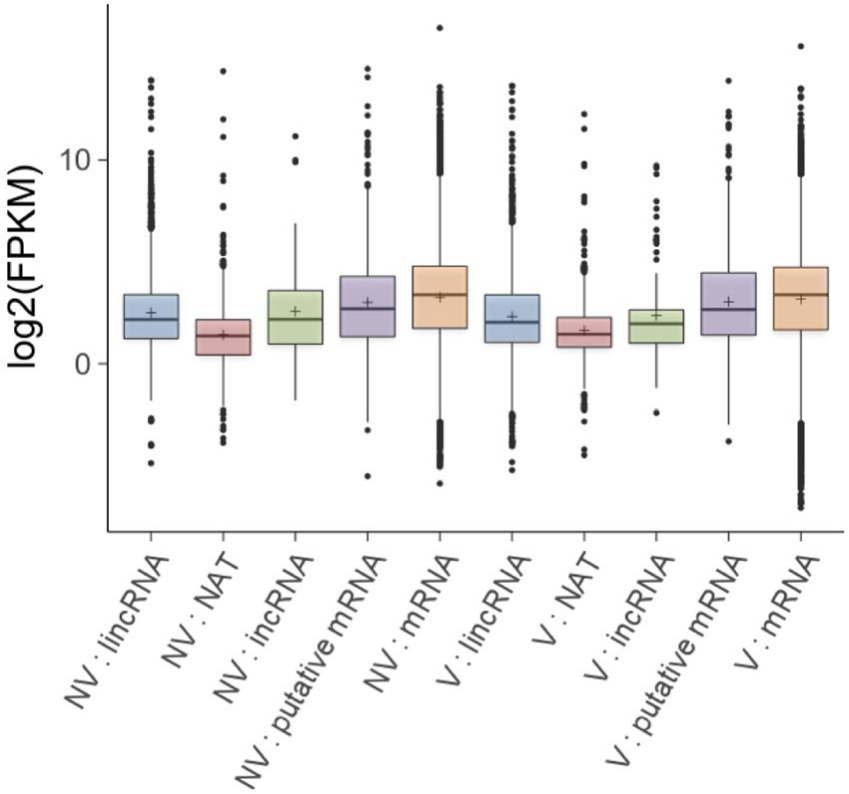
Boxplot for the expression of lincRNA, NAT, incRNA, putative mRNA, and mRNA classified transcripts prior to cold treatment (NV) and after 4 weeks of cold treatment (V). Plus (+) symbol is the mean log2 (FPKM) expression for the respective category.

We performed whole genome transcriptome analysis of first and second leaves of *B. rapa* with (V) and without (NV) four weeks of cold treatment. 2,088 lncRNAs were identified, comprised of 1,444 lincRNAs, 551 NATs, and 93 incRNAs (Supplementary Table 3) (see materials and methods). The expression levels of lincRNAs, NATs, and incRNAs were lower than those of protein-coding genes (mRNAs) in both NV and V (Tukey’s test, *p* < 0.001 for lincRNAs and NATs, *p* < 0.05 for incRNAs), and expression levels of NATs were lowest among lncRNAs in both NV and V (Fig. 2). The distribution of FPKM in the incRNAs of NV was more variable than that of V, while the distribution of FPKM in lincRNAs and NATs were similar between NV and V (Fig. 2).

We compared the expression levels of lncRNAs between NV and V. Of the 1,444 lincRNAs, 410 were differentially expressed, with 186 up-regulated and 224 down-regulated by four weeks of cold treatment (Supplementary Table 3). Of the 551 NATs, 120 were differentially expressed, with 74 up-regulated and 46 down-regulated (Supplementary Table 3). Of the 93 incRNAs, 19 were differentially expressed, with eight up-regulated and 11 down-regulated (Supplementary Table 3).

In total, thirteen lncRNAs (seven up-regulated, six down-regulated lncRNAs in V) were selected and the expression levels of these thirteen lncRNAs in NV and V were examined by qPCR. Though up-regulation of MSTRG.7460 was not confirmed by qPCR, there was a high correlation (*r* = 0.91) of the ratio of expression levels between NV and V in these thirteen lncRNAs observed between qPCR analysis and RNA-seq data (Supplementary Fig. 3).

**Figure 3:**
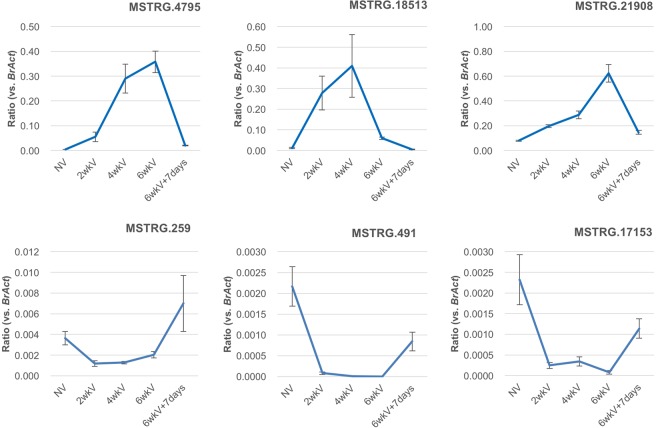
LncRNA expression levels after cold treatment measured by qPCR. Values are means ± s.e. (three biological and technical replicates) of relative expression levels compared with *Bractin*. NV, without cold treatment; 2wkV, after two weeks of cold treatment; 4wkV, four weeks of cold treatment; 6wkV, six weeks of cold treatment; 6wkV + 7 days, seven days after a return to normal growth conditions after six weeks of cold treatment.

The expression levels of six lncRNAs (up-regulated; MSTRG.4795, MSTRG.18513, MSTRG21908, down-regulated; MSTRG.259, MSTRG.491, MSTRG.17153) without cold treatment and with two, four, and six weeks of cold treatment and six weeks of cold treatment plus seven days of growth at normal temperature were examined by qPCR. The expression levels of MSTRG.4795 and MSTRG21908 increased with increasing periods of cold and their expression levels decreased on return to normal temperature (Fig. 3). Expression levels of MSTRG.18513 also increased with increasing length of cold treatment, but its expression decreased with six weeks of cold treatment. The expression levels of MSTRG. 259, MSTRG.491, and MSTRG17153 decreased following two weeks of cold treatment and were also decreased following four and six weeks of cold treatment. Their expression levels increased on return to normal temperature (Fig. 3).

### Genes differentially expressed during vernalization

Using the same RNA-seq data, we compared the expression levels of mRNA in NV and V samples and identified 8,589 differentially expressed genes (DEGs) between them, including 4,027 up-regulated and 4,562 down-regulated genes following four weeks of cold treatment (Supplementary Table 4). Genes highly up- (log2 ratio > 2.0, FDR < 0.05) or down-regulated (log2 ratio < −2.0, FDR < 0.05) by four weeks of cold treatment were categorized into GO cellular component (CC), GO molecular function (MF), and GO biological process (BP). GO categories related to stress and stimulus such as ‘Response to biotic stimulus’, ‘Response to chemical stimulus’, ‘Response to osmotic stress’ and ‘Response to salt stress’ tended to be over-represented in both up- and down-regulated genes (Supplementary Fig. 4). In up-regulated genes, GO categories of ‘Transcription factor activity’ in MF and ‘Response to heat’, ‘Response to high light intensity’, ‘Inositol biosynthetic process’ and ‘Polyol biosynthetic process’ in BP showed significant enrichment (Supplementary Fig. 4, Supplementary Table 5). In down-regulated genes, GO categories of ‘Defense response’, ‘Amine catabolic process’, and ‘Indoleacetic acid metabolic process’ in BP showed significant enrichment (Supplementary Fig. 4, Supplementary Table 5).

### Relationship between protein-coding gene and lncRNAs

**Figure 4:**
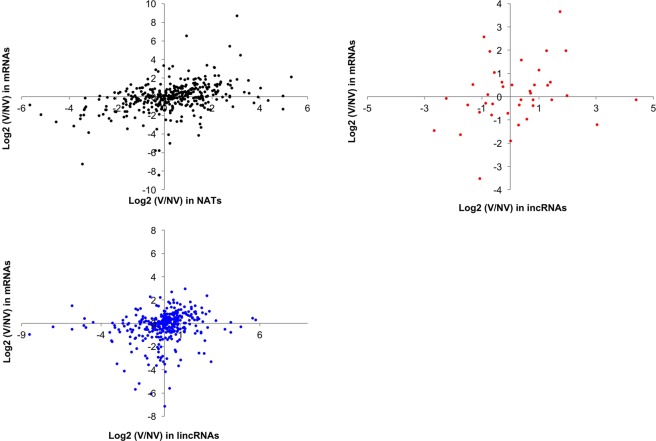
Box plot representing the change of expression levels of lncRNAs and their paired mRNAs by four weeks of cold treatment. In the case of lincRNAs, the genes within 2 kb from lincRNAs were used for comparison. V, with four weeks of cold treatment; NV, without cold treatment.

To examine whether the change of lncRNAs is associated with the change of expression level of mRNAs covering or close to lncRNAs, we examined the correlation coefficient between ratios of the expression change of NV and V (V/NV ratio) in mRNA and incRNA pairs and in mRNA and NAT pairs. There was no association in the V/NV ratio between mRNA and paired incRNAs or between mRNA and paired NATs (Fig. 4), suggesting that the majority of transcriptional changes of mRNA expression by four weeks of cold treatment is independent of the expression of their paired incRNAs or NATs. We also examined the correlation coefficient of the V/NV ratio between mRNA and lincRNAs, which are located within 2 kb distance of mRNAs, and there was no correlation between them (Fig. 4).

**Figure 5:**
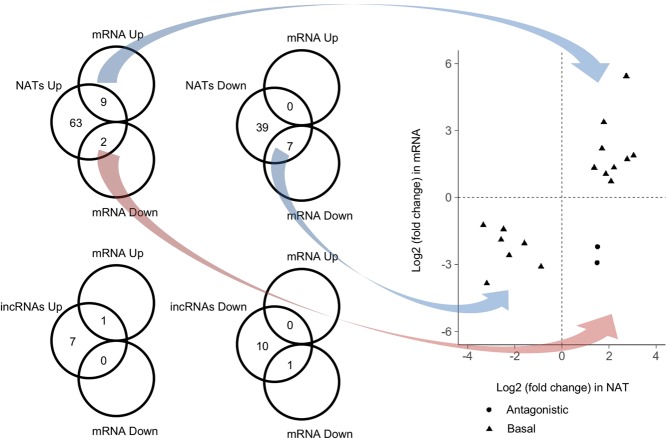
The relationship of transcriptional changes by four weeks of cold treatment between mRNA and paired incRNAs or NATs. Plot of log2 score of fold change of FPKM in V (four weeks of cold treatment) divided by that in NV (before cold treatment) in NAT and mRNA pairs expression, showing the four possible expression patterns as illustrated by the Venn diagrams on the left. The lower two Venn diagrams show the observed expression patterns for mRNAs containing incRNAs. mRNAs showing shared up- or down-regulation with their respective NAT transcript are shown as triangles. mRNAs showing opposing up- or down-regulation in comparison to their respective NAT transcript are shown as circles.

We focused on the genes covering up- or down-regulated NATs or incRNAs following four weeks of cold treatment. Of 74 mRNAs covering up-regulated NATs, nine genes showed up-regulation and two genes showed down-regulation (Table 1, Fig. 5). Of 46 mRNA covering down-regulated NATs, seven genes showed down-regulation by four weeks of cold treatment (Table 1, Fig. 5). In total, 16 genes showed coordinate gene expression pattern with NATs and only two genes showed antagonistic gene expression patterns by four weeks of cold treatment (Table 1, Fig. 5).

**Table 1:** Differentially expressed NATs or incRNAs by four weeks of cold treatment.

	Expression level of lncRNAs (FPKM)		Fold change	Paired mRNAs	Expression level of mRNAs (FPKM)		Fold change		Orthologs in *A. thaliana*	Gene description
	NV	V	log2 (V/NV)	ID	NV	V	log2(V/NV)			
**NATs**										
**Up-regulated**										
MSTRG.2161	0.49	4.04	3.05	Bra021594	18.91	69.48	1.88	Up	AT3G15210	*ERF4*
MSTRG.10467	0.88	6.04	2.77	Bra004446	0.89	2.93	1.71	Up	AT3G62690	*ATL5*
MSTRG.4795	1.83	12.20	2.74	Bra029292	1.35	58.72	5.44	Up	AT5G62020	*HSFB2A*
MSTRG.23036	1.05	4.86	2.21	Bra007504	4.02	10.18	1.34	Up	AT3G60080	*RING/U-box superfamily protein*
MSTRG.3577	0.93	3.94	2.09	Bra039752	7.26	11.98	0.72	Up	AT1G66160	*CMPG1*
MSTRG.5770	0.45	1.64	1.87	Bra006862	1.08	2.25	1.05	Up	AT5G01560	*LECRKA4.3*
MSTRG.20836	0.88	3.03	1.78	Bra037845	2.57	26.69	3.38	Up	AT5G67450	*ZF1*
MSTRG.8355	1.45	4.71	1.70	Bra024126	43.12	197.36	2.19	Up	AT4G29780	*Nuclease*
MSTRG.15105	2.80	7.26	1.37	Bra025323	7.79	19.67	1.34	Up	AT3G28180	*CSLC4*
MSTRG.7460	5.92	16.90	1.51	Bra001846	31.46	6.81	−2.21	Down	AT3G21760	*HYR1*
MSTRG.21908	8.75	24.69	1.50	Bra029572	7.07	0.93	−2.92	Down	AT4G05330	*AGD13*
**Down-regulated**										
MSTRG.2598	7.53	4.08	−0.88	Bra028893	14.69	1.71	−3.10	Down	AT5G01210	*HXXXD-type acyl-transferase family protein*
MSTRG.17623	9.66	3.21	−1.59	Bra016256	41.96	10.07	−2.06	Down	AT1G69890	*Actin cross-linking protein*
MSTRG.7151	9.17	1.94	−2.24	Bra001390	44.61	7.40	−2.59	Down	AT3G11110	*RING/U-box superfamily protein*
MSTRG.25903	4.79	0.86	−2.48	Bra009516	9.37	3.51	−1.42	Down	AT5G03700	*D-mannose binding lectin protein with Apple-like carbohydrate-binding domain-containing protein*
MSTRG.17356	8.32	1.39	−2.58	Bra004009	34.78	9.35	−1.89	Down	AT1G69050	*Hypothetical protein*
MSTRG.17011	2.65	0.29	−3.19	Bra003511	4.48	0.31	−3.85	Down	AT3G62830	*AUD1*
MSTRG.5884	16.78	1.66	−3.34	Bra029099	11.87	5.02	−1.24	Down	AT5G53050	*Alpha/beta-Hydrolases superfamily protein*
**incRNAs**										
**Up-regulated**										
MSTRG.14559	119.61	251.26	1.07	Bra024395	1.54	17.65	3.52	Up	AT5G65530	*Protein kinase superfamily protein*
**Down-regulated**										
MSTRG.1863	10.94	3.29	−1.73	Bra023842	4.73	0.38	−3.65	Down	AT3G22240	*Cysteine-rich/transmembrane domain PCC1-like protein*

Of eight mRNAs covering up-regulated incRNAs, one gene showed up-regulation by four weeks of cold treatment (Table 1, Fig. 5). Of 11 mRNAs covering down-regulated incRNAs by four weeks of cold treatment, one gene showed down-regulation by four weeks of cold treatment (Table 1, Fig. 5). Overall, no gene showed antagonistic expression patterns with incRNAs by four weeks of cold treatment, while two genes showed coordinate expression patterns (Table 1, Fig. 5).

These results indicate that for most genes, change of NAT or incRNA expression levels does not affect the expression level of the mRNAs covering them. When the expression levels of mRNA did change, it tended to be coordinate rather than antagonistic.

### NATs derived from *FLC* or *MAF* loci had increased expression with short-term cold treatment

**Figure 6:**
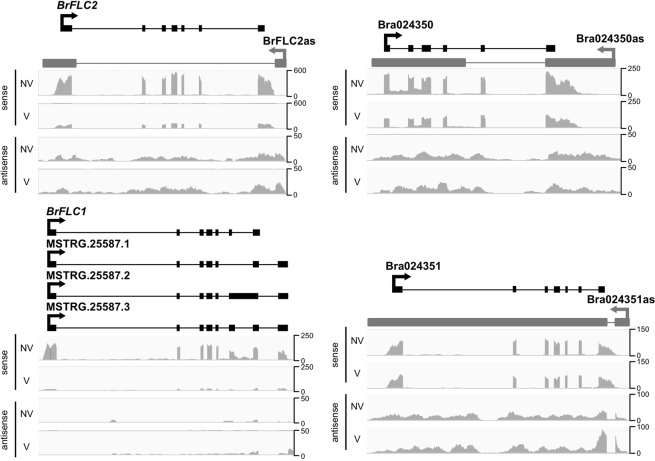
Strand-specific read coverage plots for *BrFLC2*, *BrFLC1*, Bra024350, and Bra024351. Above each coverage plot, the mRNA transcripts are shown in black and the antisense transcripts (NATs) are shown in gray, with exons represented as thick bars and an arrow showing the 5′ to 3′ sense, and thin lines illustrating the introns. For *BrFLC1*, three splice isoforms are shown relative to the reference genome annotation, illustrating a conservation of mRNA structure for exons 1 through 5, with differential splicing occurring at exons 6 and 7.

From the RNA-seq data, three *FLC*s, *FLC1* (Bra009055), *FLC2* (Bra028599), and *FLC3* (Bra006051), were down-regulated by four weeks of cold treatment (Supplementary Table 4). As three cold-responsive noncoding RNAs (COOLAIR^3^, COLDAIR^4^, and COLDWRAP^5^) were reported within the *FLC* locus in *A. thaliana*^10,11^, we examined the cold-responsive noncoding RNAs in *B. rapa*. Of the *BrFLC* loci (*BrFLC1*, *BrFLC2*, and *BrFLC3*), only *BrFLC2* contained a NAT, termed BrFLC2as (MSTRG.2765) (Fig. 6), which has a similar structure to COOLAIR of *A. thaliana*. No transcripts homologous to the *A. thaliana* lncRNA sequences COLDAIR and COLDWRAP were found at any of the *FLC* loci in *B. rapa* (Fig. 6). We found splicing variants in *BrFLC1* mRNAs, though there were no lncRNAs at the *BrFLC1* locus (Fig. 6).

**Figure 7:**
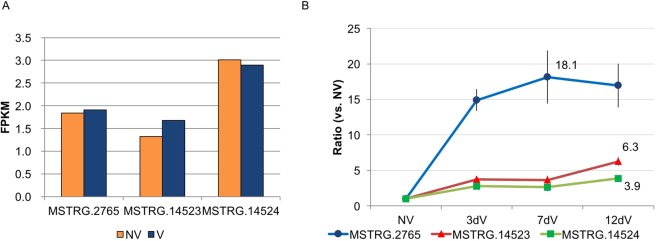
Expression levels of three NATs before and after cold treatment. (**A**) RNA-seq expression (FPKM) for three NATs before (NV) and after (V) four weeks of cold treatment. Differential expression analysis showed that none of the three NATs were differentially expressed at the FDR <0.05 level. (**B**) qPCR results showing expression ratios normalized to NV for three NATs prior to cold treatment (NV), three, seven, and twelve days into cold treatments (3dV, 7dV, and 12dV, respectively). BrFLC2as (MSTRG.2765), *MAF1* homolog Bra024350as (MSTRG.14523), *MAF4* homolog Bra024351as (MSTRG.14524).

We also identified down-regulation of two (Bra024350, Bra031888) of five *MAF* genes by four weeks of cold treatment (Supplementary Table 4, Supplementary Fig. 5). The down-regulated *MAF* genes in *B. rapa* were both *AtMAF1* homologs (Supplementary Fig. 5). Bra024350 contained a NAT, Bra024350as (MSTRG.14523) (Fig. 6). However, another *AtMAF1* homolog Bra031888 did not have a NAT (Supplementary Fig. 5). Like *AtMAF1*, *AtMAF4* has two homologs (Bra024351 and Bra031884) that were not down-regulated by four weeks of cold treatment in *B. rapa* (Supplementary Fig. 5). Bra024351 contained a NAT, Bra024351as (MSTRG.14524) (Fig. 6), while no NAT was found for Bra031884 (Supplementary Fig. 5). None of these three NATs was differentially expressed between NV and V (FDR < 0.05) (Fig. 7A). On the other hand, qPCR analysis of BrFLC2as and the two MAF NATs (Bra024350as and Bra024351as) revealed that BrFLC2as transcription was induced during the initial onset of cold treatment, whereas the two MAF NATs showed only a slight increase in transcription in response to the onset of cold (Fig. 7B).

Examination of the BrFLC2as sequence, by alignment with ClustalW, to the corresponding *BrFLC1* and *BrFLC3* anti-sense sequences did not show any sequence similarity; and alignment of the 5′ 350 bp upstream sequence to the corresponding anti-sense regions at the *BrFLC1* and *BrFLC3* loci also had no sequence homology, suggesting that the BrFLC2as is specific to the *BrFLC2* locus. The BrFLC2as transcript differs slightly in length from the full-length *A. thaliana* COOLAIR sequence, with the BrFLC2as transcript having a length of 671 bp, as compared to the 558 bp long (poly-A tail removed) COOLAIR sequence. Alignment of the two sequences with ClustalW to the anti-sense strand of the *BrFLC2* locus (with 500 bp flanking regions) revealed the COOLAIR sequence was 78.4% identical along a 571 bp aligned region, consistent with the corresponding sense strand’s conserved homology of the *AtFLC* and *BrFLC2* exon 1 coding region and the 5′ UTR region (Supplementary Fig. 6). However, the 5′ ends of the BrFLC2as and COOLAIR sequences show little homology (<30% identity), suggesting that the promoter region of the BrFLC2as sequence differs from that of *A. thaliana* COOLAIR.

## Discussion

We studied the transcriptional response of lncRNAs following vernalization in *B. rapa* as a number of lncRNAs have been reported to be critical for vernalization in *A. thaliana*. Previous studies have shown that some lncRNA alter expression in response to abiotic stresses^28–33^, suggesting that transcriptional changes of lncRNAs may play a role in stress response. We identified 2,088 lncRNAs in total, comprised of 1,444 lincRNAs, 551 NATs, and 93 incRNAs of which 549 significantly altered their expression in response to four weeks of cold treatment. The expression levels of some lncRNAs were altered in response to the different durations of cold treatment. Some lncRNAs increased or decreased their expression levels, but rapidly returned to a non-vernalized level upon return to normal growth conditions.

We also identified the DEGs between NV and V. GO categories related to stress or stimulus tended to be overrepresented in both up- and down-regulated genes; e.g., ‘Response to cold’ category was significantly overrepresented in both up- and down-regulated mRNAs. The cold responsive gene *BrCOR15B* was highly induced in response to cold treatment, but rapidly returned to previous expression levels upon a return to normal growth conditions. These results suggest that the differentially expressed lncRNAs or mRNAs detected in this study are mixed, with some involved in the vernalization response and others involved in the physical cold response, possibly in the form of DNA conformational changes that alter the transcriptional patterns of the genome and possibly help initiate the cold response^34^. Therefore, changes of lncRNA or mRNA expression are due to either the vernalization response (long-term cold), the cold response including short-term cold, or merged effects. This study could not distinguish these differences, thus further detailed analysis will be required with different durations of cold treatments in order to clarify the differences in the effect of short and long-term cold response.

We investigated the relationship between lncRNAs and mRNA expression with the corresponding NAT expression or incRNA expression in response to four weeks of cold treatment. There was little association of changed expression level by four weeks of cold treatment between mRNAs and paired lncRNAs (either within genic regions or close to genes), suggesting that the transcriptional response of mRNA and the paired lncRNAs is independently regulated. Some differentially expressed mRNAs, which had a differentially expressed NAT or incRNA altered expression in the same direction. There were few pairs of differentially expressed mRNA and NATs or incRNAs showing antagonistic expression patterns. In *B. rapa*, there was mostly a positive connection between mRNA and lncRNA in cold and heat stresses, consistent with some genes being regulated by lncRNAs^35^. In *A. thaliana*, protein coding *cis*-NATs are broadly expressed under various stress conditions with high co-expression, suggesting that one of the functions of overlapping gene-NAT pairs is to promote high and broad gene transcription^36^. In this study, half of the genes showing co-upregulation of mRNA and their corresponding NATs were stress-responsive, such as *ERF4*, *HSFB2A*, *CMPG1*, *LECRKA4.3*, and *AZF1* in *B. rapa*. In *A. thaliana*, both *HSFB2a* and *asHSFB2a*, which is transcribed in an opposite direction starting from 3’-end of the *HSFB2a*, were induced by heat stress^37^. We suggest that the co-expression of mRNA-NAT pairs is involved in abiotic stress response.

COOLAIR has been suggested to regulate the initial response of *FLC* during vernalization in *A. thaliana*^3^. We found COOLAIR-like transcripts at the *BrFLC2* locus (BrFLC2as), while there was no COOLAIR-like transcript found for the other three *BrFLC* paralogs. Like COOLAIR in *A. thaliana* BrFLC2as was up-regulated by short-term cold treatment. The COOLAIR sequence found in *A. thaliana* appears to have a different structure from that of the BrFLC2as sequence. Because COOLAIR appears to function as a *cis*-NAT with respect to the *AtFLC* locus^38^, the specificity of BrFLC2as to the *BrFLC2* locus raises two distinct possibilities with respect to its function. If BrFLC2as acts in *cis*, then it does not regulate the expression of *BrFLC1* and *BrFLC3*, both of which are important in the vernalization of *B. rapa*^11,17^. On the other hand, if BrFLC2as acts in *trans*, the function of BrFLC2as is markedly different from that of COOLAIR. *BrFLC2* specific antisense transcripts have been identified and the over-expression of *BrFLC2* NATs resulted in a weaker vernalization requirement and the transcriptional suppression not only of *BrFLC2* but also of *BrFLC1* and *BrFLC3*, suggesting that *BrFLC2* NATs are involved in the suppression of *BrFLC* paralogs in *trans* during vernalization^19^. However, this result could not distinguish between endogenous epigenetic silencing of *BrFLC* paralogs with H3K27me3 accumulation by vernalization and post-transcriptional gene silencing by expressing an antisense transgene.

COOLAIR-like NATs are conserved in species related to *A. thaliana* as well as in *B. rapa*, hinting at an important biological function including the possibility of the involvement in the repression of *FLC* by cold treatment^39^. However, whether COOLAIR is involved in *FLC* silencing remains controversial^40,41^. There is no evidence that COOLAIR is involved in recruitment of the PRC2 complex to the *FLC* locus whereas the cold-inducible COLDAIR and COLDWRAP, which are transcribed from the *AtFLC* first intron and from the promoter in the sense direction, respectively, have been shown to be involved in PRC2 recruitment to *AtFLC* by cold treatment^10,11^. In the perennial species *A. alpina*, COLDAIR-like transcripts were not identified at the *PEP1* locus (an ortholog of *FLC*), but transcription was silenced by vernalization^39^. However, this transcriptional silencing was not maintained^39^. In *B. rapa*, we have confirmed the accumulation of H3K27me3 in all *BrFLC* paralogs by vernalization and transcriptional silencing was maintained upon returning to warm condition after four weeks of cold treatment^18^, suggesting that the PRC2 complex is recruited in all *BrFLC* paralogs and induces stable epigenetic repression^18^. The lack of evidence for COLDAIR and COLDWRAP transcripts in our results raises another important question regarding the molecular mechanisms of vernalization in *B. rapa*. Without COLDAIR and COLDWRAP transcripts, it is not clear how H3K27me3 accumulation is mediated and raises questions regarding the role of noncoding RNAs in the vernalization of *B. rapa*. Further study will be needed to clarify this question.

In this study, we found NATs in two paralogs of *MAF* genes, Bra024350 (*AtMAF1*-like) and Bra024351 (*AtMAF4*-like), in *B. rapa*. Both NATs were slightly up-regulated by short-term cold treatment. The *AtMAF1*-like genes, Bra024350 and Bra031888, were silenced by four weeks of cold treatment, while the *AtMAF4*-like genes, Bra024351 and Bra031884, were not. In one *AtMAF1*-like gene, Bra024350, H3K27me3 was accumulated during four weeks of cold treatment and H3K27me3 levels increased upon a return to warm conditions after cold treatment^18^. An *MAF*-like gene, *BcMAF1*, has been shown to act as floral repressor in *B. rapa*^42^. In *A. thaliana*, *MAF* genes were silenced by cold treatment, and accumulation of H3K27me3 was observed in some *MAF* genes. There is no report of cold induced noncoding RNAs at the *MAF* loci of *A. thaliana*. At least in *B. rapa*, the PRC2 complex could be recruited to *MAF* loci after four weeks of cold treatment whether there are cold induced noncoding RNAs or not, similar to the *BrFLC* paralogs. These results suggest that the noncoding RNAs found in *B. rapa* may function in a different manner to noncoding RNAs in the vernalization of *A. thaliana*.

## Supplementary information

Supplementary Figures

Supplementary Tables

## Data Availability

The data have been deposited with links to SRA accession number SRP156464 in the NCBI SRA database (https://www.ncbi.nlm.nih.gov/sra/).
