# Supplementary material for: Long noncoding RNAs in Brassica rapa L. following vernalization
Source: Sci Rep. 2019 Jun 26;9:9302. doi: 10.1038/s41598-019-45650-w (PMC6594933; doi:10.1038/s41598-019-45650-w)
Supplement: Supplementary file 1 — Supplementary Figures [file 41598_2019_45650_MOESM1_ESM.pdf]

Supplementary Information

Title of the manuscript:

Long noncoding RNAs in *Brassica rapa* L. following vernalization

Author list:

Daniel J. Shea, Namiko Nishida, Satoko Takada, Etsuko Itabashi, Satoshi Takahashi, Ayasha Akter, Naomi Miyaji, Kenji Osabe, Hasan Mehraj, Motoki Shimizu, Motoaki Seki, Tomohiro Kakizaki, Keiichi Okazaki, Elizabeth S. Dennis, Ryo Fujimoto

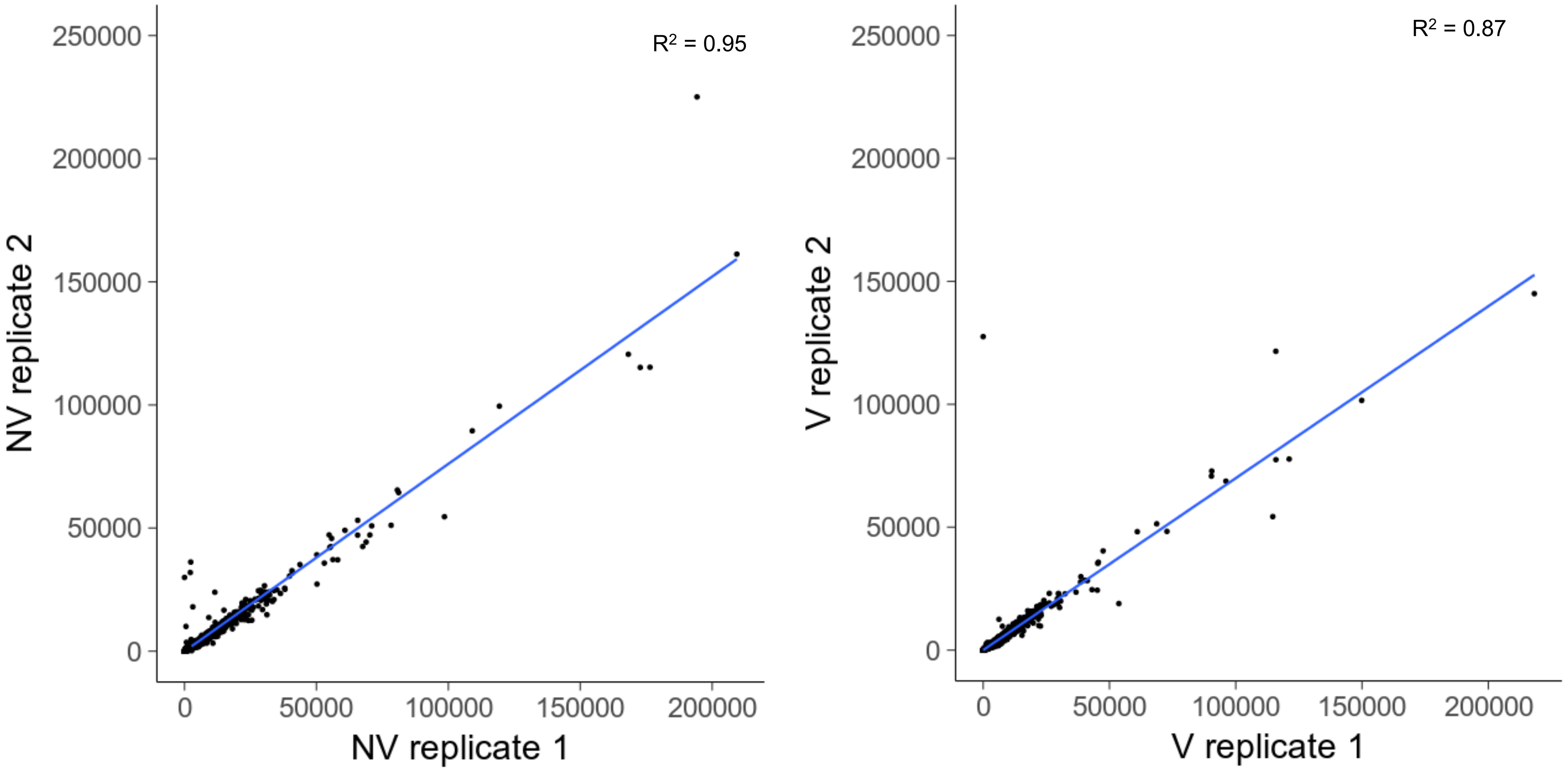

Supplementary Figure 1. Correlation between replicates for RNA-seq data. The plots show pairwise gene tracking transcript counts for samples taken prior to cold treatment (NV) and samples taken after four weeks of cold treatment (V). The coefficient of determination (R^2) was 0.95 for the NV samples, and 0.87 for the V samples, respectively.

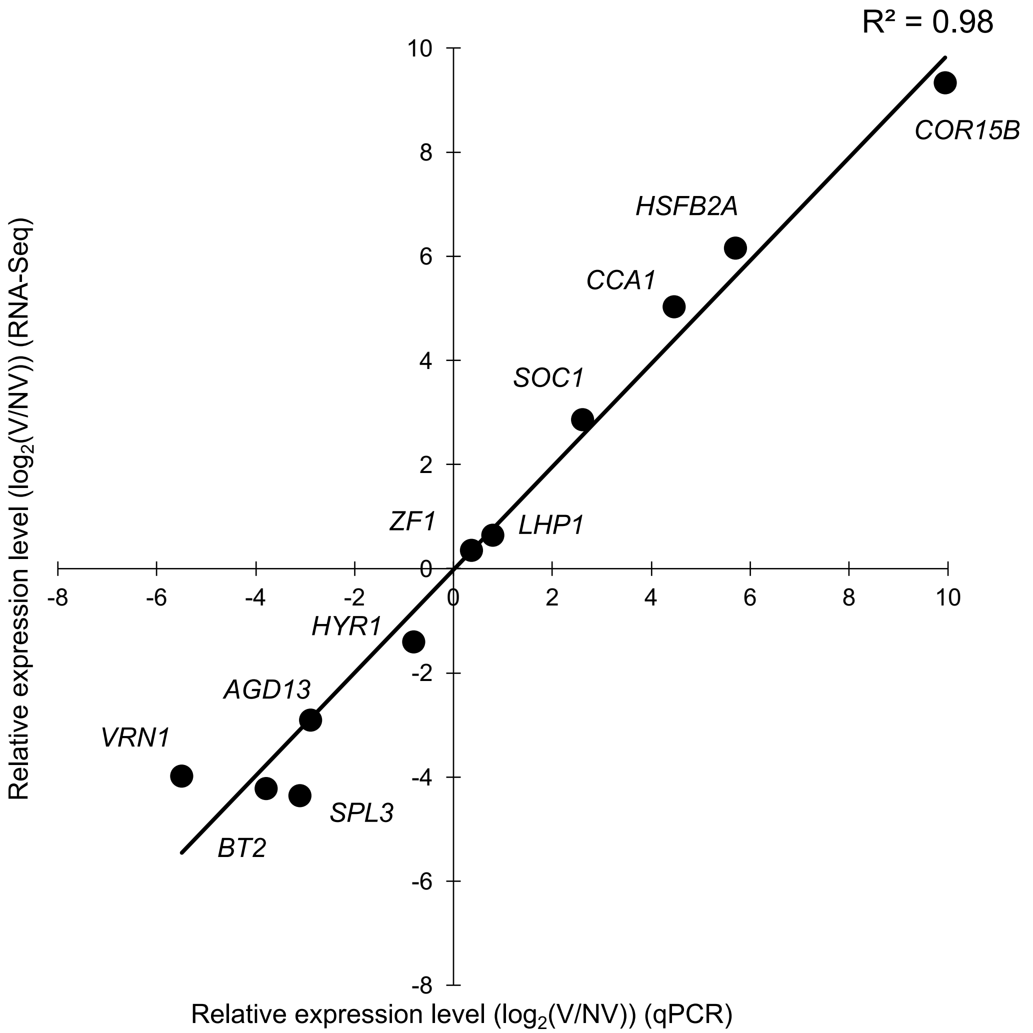

Supplementary Figure 2. Pairwise plotting of relative expression levels for eleven genes examined by qPCR and RNA-seq. The coefficient of determination was calculated to be 0.98. The qPCR expression levels for both no cold treatment (NV) and four weeks of cold treatment (V) conditions are normalized to *Bractin*.

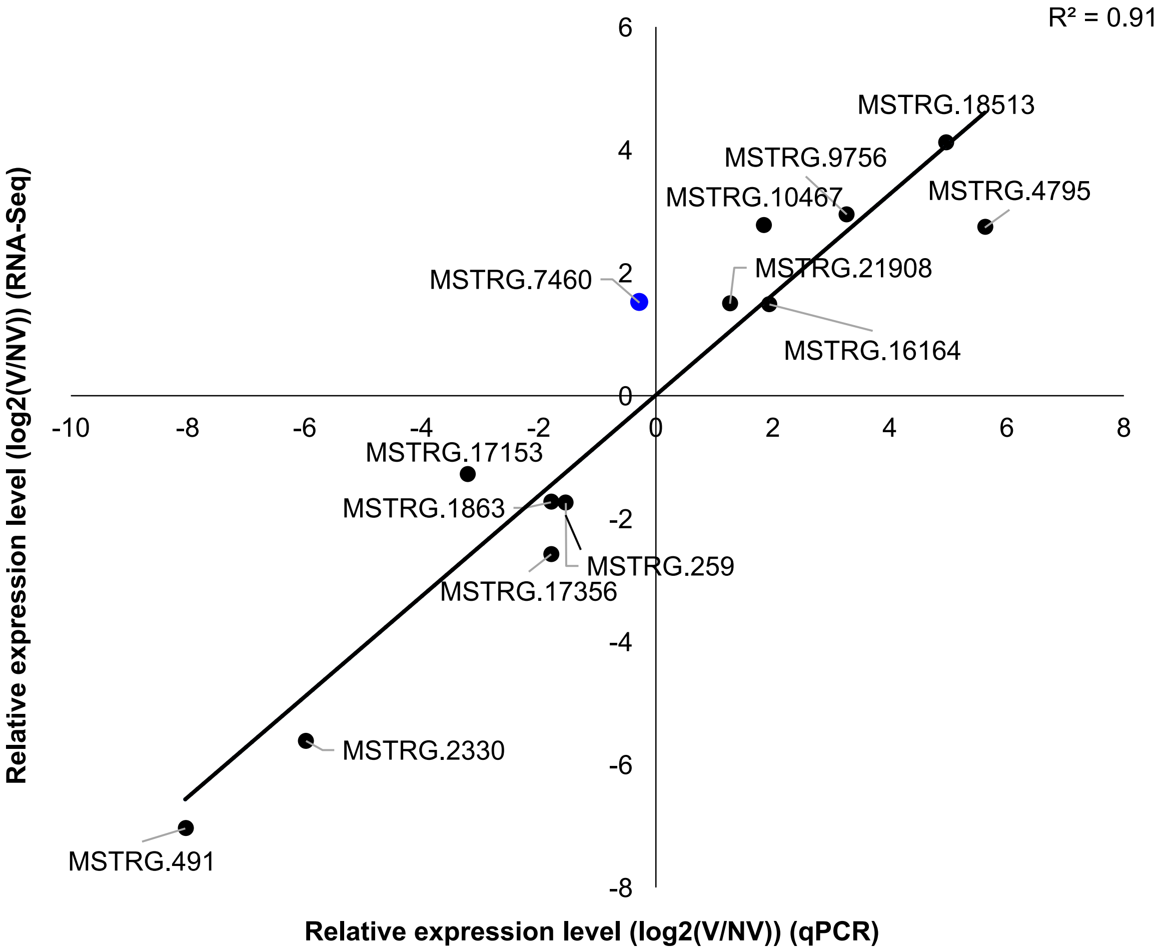

Supplementary Figure 3. Pairwise plotting of relative expression levels for thirteen NAT transcripts examined by qPCR and RNA-seq. The coefficient of determination was calculated to be 0.91. The blue data point for MSTRG.7460 shows that up-regulation of the transcript was not confirmed by qPCR. The qPCR expression levels for both no cold treatment (NV) and four weeks of cold treatment (V) conditions are normalized to *Bractin*.

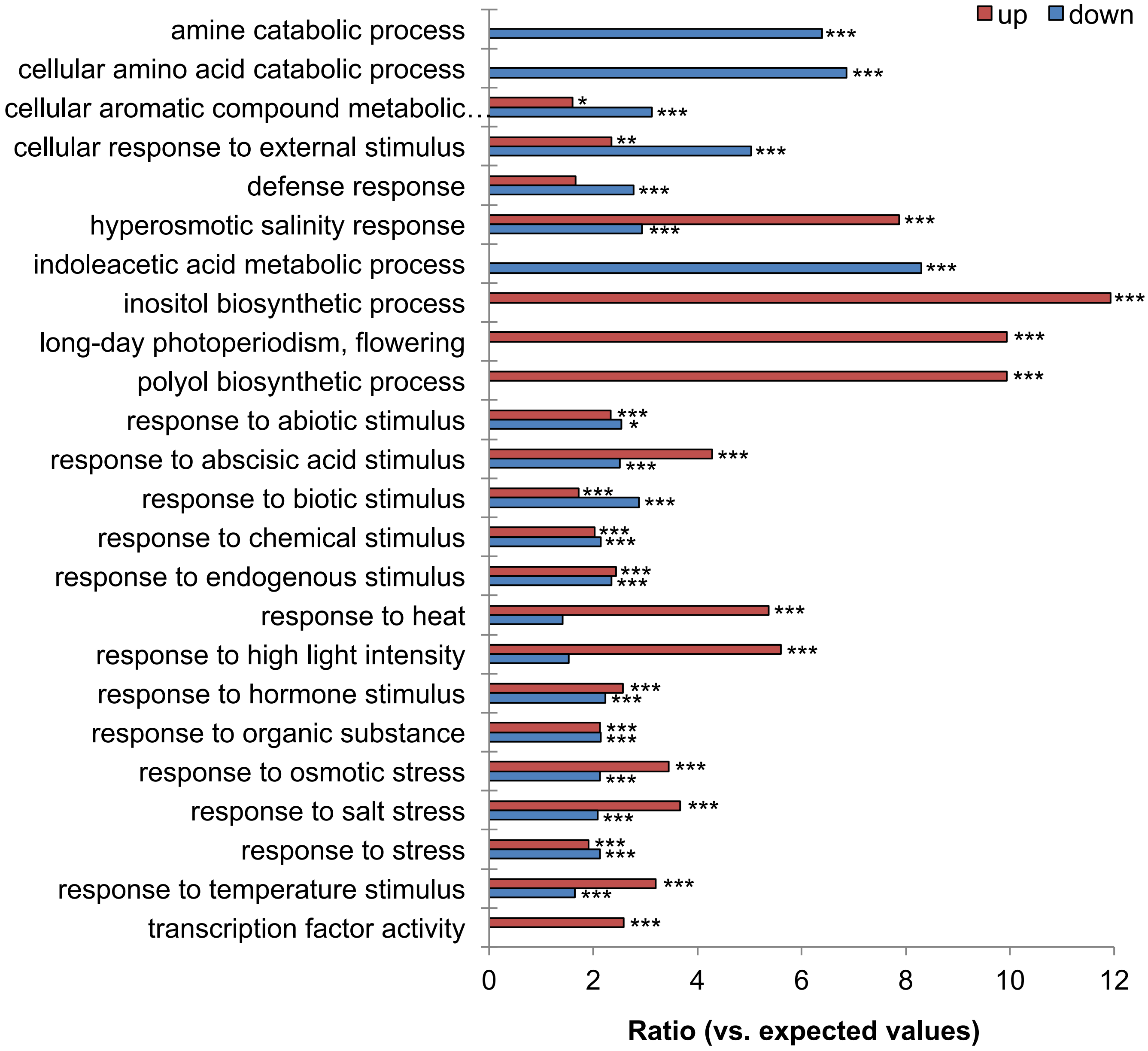

Supplementary Figure 4. Gene ontology (GO) classification of highly up- (log2 ratio > 2.0, FDR < 0.05) and down- (log2 ratio < -2.0, FDR < 0.05) regulated genes as a result of four weeks of cold treatment. *, $p < 0.05$; **, $p < 0.01$; ***, $p < 0.001$.

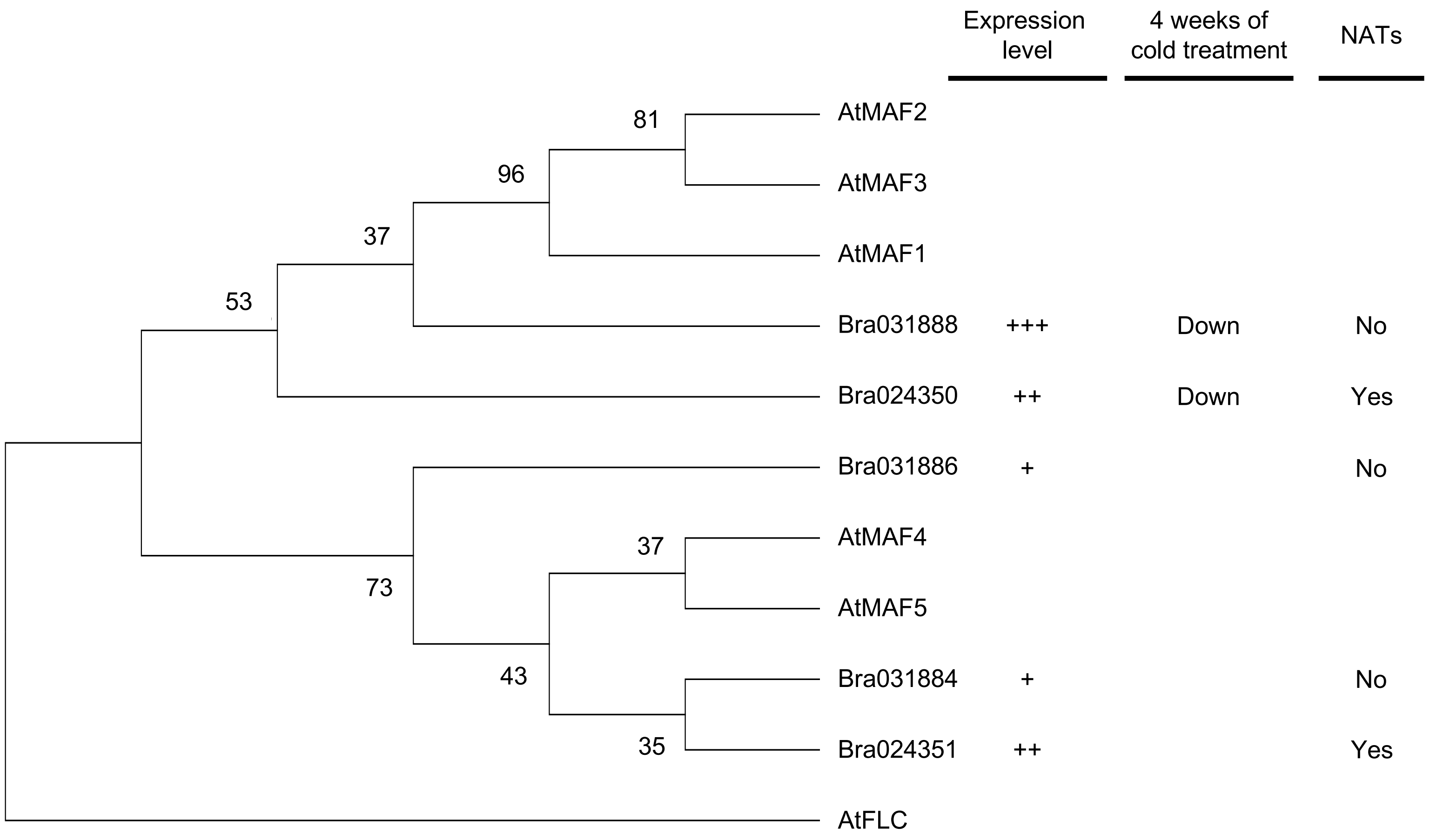

Supplementary Figure 5. Phylogenetic tree of amino acid sequences of *MAF* genes in *A. thaliana* and *B. rapa* using the Maximum Likelihood method based on the Tamura 3-parameter model. The bootstrap values with 10,000 replicates represent at the node of phylogenetic tree. Initial tree(s) for the heuristic search were obtained automatically by applying Neighbor-Join and BioNJ algorithms to a matrix of pairwise distances estimated using the Maximum Composite Likelihood (MCL) approach, and then selecting the topology with superior log likelihood value. The rate variation model allowed for some sites to be evolutionarily invariable ([+I], 35.9905% sites). The analysis involved 11 nucleotide sequences. All positions containing gaps and missing data were eliminated. There were a total of 170 positions in the final dataset. The expression levels of *MAF* genes in *B. rapa* were categorized into three levels with increasing plus numbers showing higher expression levels. ‘Down’ represents the expression levels that were significantly downregulated by four weeks of cold treatment.

```
MSTRG.2765  TGGCCACGT-CTCTCTCTACTGCAAATAAGGTAGTTCTTCTGTCTTCACCATGAAGCAAACACGAGTTTTGACAGAAG-AGACGAACACACACAGACAAAC
COOLAIR     -----ACATACACTCAAGATCTCGAT----GCAATTCTC--------------------ACACGAAT---AAGAAAAGTAAAAGAGCACA-------AAAC
                 ** * * ***   *   * *     * * ****                     ****** *    * * *** * * ** ****       ****

MSTRG.2765  ACAATCTTTTGACAGAAGAGACTAACACACACAAACTCTCTACGATTCTCTCTCAAAGAAGGCGGTACACGTGGCTGTCTTGTCCCTGAACCACATTGGTT
COOLAIR     AGAA------GATAAAAGGGGG-AACAAATGAAAACCCA----GGT---------AAGGAAAAGGCGTAC---------TTATCGCCGGAGGA--------
            * **      ** * *** *   **** *   **** *     * *         *** *   **   **         ** ** * * *  *

MSTRG.2765  CTTTCTACGAATTTTTATTTTCATCTCTCTCGTTTACCCTAATAAAAAGTGGCCCGAGGGAGAAAAAGGAGAGACACAAAAAGAAAGAAATAAAAGCAAAA
COOLAIR     --------GAAGCTGTAGA------------GCTTGCCGGAGGCGGAGACGAC------GAGAAGAGCGACGGATGCGTCAC-AGAGAACAGAAAGCTGAC
                    ***  * **              * ** **  *     *   * *      ***** *  **  **  *   *  * ****   *****  *

MSTRG.2765  AAG------AAAGAAAATAAAAGCAAAAATAAGAAAGAACAAAAAACGCTCAGTATCTCCGGCGAGAGTTGAAACCGAACCTCAGGATCAAATTAGGGCAC
COOLAIR     GAGCTTTCTCGATGAGACCGTTGCGACGTTTGGAGAAGGTGACT--TGTCGGCTACTTTTGTTCTCAATTCG--CTTGATTTCTAGTTT---TTTTTTTCC
             **        *  * *     ** *   *  ** *     *     *     **  *  *     * **    *   *  **  * *    **      *

MSTRG.2765  AAAGGCTTCTCGGAGACAGAAGCCATGGGAAGAAAGAAACTAGAGATCAAGCGAATTGAGAACAAAAGTAGCCG-----ACAAGTCACCTTCTCCAAACGA
COOLAIR     CATGGCTTCTCTCCGAGAGG-GCTTTGTGCCCTAAT---TTGATCCTCAGGT---TTGGGTTCAA--GTCGCCGGAGATACTAAGCGTTTTCTCT------
             * ********   ** **  **  ** *    **     *     *** *    *** *  ***  ** ****     ** *  *   *****

MSTRG.2765  CGCAATGGTCTCATCGAGAAAGCTCGTCAGCTTTCAGTTCTCTGCGATGCATCCGTCGCTCTTCTCGTTGTCTCAGCCTCCGGCAAGCTTTACAACTTCTC
COOLAIR     TTCTATTTTTTTTTTTC-----CTTTTCTCGCTTTATTTCTTTCTATTTTTTGTGCCTATCTACT--TTTTCTTCG--TCGGGCCAGATATTT--TTTTTT
              * **  * *  *        **  **    ** * **** *    *   *  * *  *** **  ** ***  *  ** *** ** * *     ** *

MSTRG.2765  CGCCGGCGATAAGTATGACTTCT---------CCTCTTCTGGGTCT-----TCCTTTATTT----GCCCTTTTCGCTTC-CGTTCCTCTTCTCTTTA
COOLAIR     TTTTGGGGGTAAACGAGAGTGATGCAAAAAAACCAAATATGTGAATAAAAACGTTGTGTTTTGAAGACAAGATTGCCACGTGTACCGC---------
                ** * ***    ** *  *         **   * ** *       *   * * ***    * *    * **  *  ** ** *
```

Supplementary Figure 6. Clustalw alignment of the BrFLC2as (shown here as MSTRG.2765) transcript to the *A. thaliana* COOLAIR sequence at the 5’ UTR region. Blue and purple letters represent exon 1 and exon 2, respectively.
